# Effects of live-remote exercise on quality of life and other health-related outcomes in cancer survivors: a randomised controlled trial

**DOI:** 10.1007/s11764-025-01845-x

**Published:** 2025-06-05

**Authors:** Melissa Kotte, Kate A. Bolam, Renske Altena, Prue Cormie, Yvonne Wengström, Sara Mijwel

**Affiliations:** 1 Department of Neurobiology, Care Sciences and Society, Karolinska Institutet, Stockholm, Sweden; 2 Cardiometabolic Health and Exercise Physiology Lab, Baker Heart and Diabetes Institute, Melbourne, Australia; 3 Department of Physical Activity and Health, The Swedish School of Sport and Health Sciences, Stockholm, Sweden; 4 Department of Cardiometabolic Health, The University of Melbourne, Melbourne, Australia; 5 School of Health, University of the Sunshine Coast, Sunshine Coast, Australia; 6 Department of Oncology-Pathology, Karolinska Institutet, Stockholm, Sweden; 7 Peter MacCallum Cancer Centre, Melbourne, Australia; 8 Sir Peter MacCallum Department of Oncology, The University of Melbourne, Melbourne, Australia; 9 Karolinska Comprehensive Cancer Centre, Theme Cancer Karolinska University Hospital, Stockholm, Sweden

**Keywords:** Virtual exercise, Home-based exercise, Telehealth, Cancer, HRQoL

## Abstract

**Purpose:**

Exercise following cancer treatment has been shown to improve health-related quality of life (HRQoL) and other important health outcomes, yet barriers to participation prevent many cancer survivors from exercising according to recommendations. Live-remote exercise methods could improve accessibility; however, evidence on their efficacy is limited. The EX-MED Cancer Sweden randomised controlled trial compared the effects of a 12-week live-remote online exercise intervention to usual care in adult cancer survivors.

**Methods:**

Two hundred adults who had completed curative treatment for breast, prostate, or colorectal cancer were randomised to a 12-week live-remote online exercise intervention or a usual care control group. Intervention effects on the primary outcome overall HRQoL (EORTC-QLQ-C30) and secondary outcomes cardiorespiratory fitness (CRF), muscle strength, physical function, body composition, and other patient-reported outcomes were determined by comparing changes from baseline to 3 (primary timepoint) and 6 months between groups.

**Results:**

No significant effect was observed on overall HRQoL. However, live-remote exercise resulted in significant improvements at 3 months on the physical functioning domain of HRQoL (*p* ≤ 0.001), five-times sit-to-stand (*p* = 0.003), and moderate-vigorous physical activity levels (*p* ≤ 0.001) as well as estimated VO_2 max_ (*p* = 0.045), and upper body strength (*p* = 0.010) at 3 and 6 months. No significant differences were observed between the groups on lower body strength, handgrip strength, fatigue, or the other functional domains or symptoms of the EORTC-QLQ-C30.

**Conclusions:**

A 12-week live-remote exercise intervention did not lead to improvements in overall HRQoL; however, it did result in significant benefits in physical function, CRF, and upper body strength in adults treated for breast, prostate, or colorectal cancer. These findings add to the limited evidence on the effects of virtually supervised exercise for cancer survivors.

**Implications for Cancer Survivors:**

EX-MED Cancer Sweden addresses common exercise barriers for cancer survivors while providing the benefits of supervised exercise.

**Trial registration:**

NCT05064670, Trial registered on October 1, 2021, https://clinicaltrials.gov/ct2/show/NCT05064670

**Supplementary Information:**

The online version contains supplementary material available at https://doi.org/10.1007/s11764-025-01845-x.

## Introduction

As a result of advancements in early detection and treatment, there are 32 million cancer survivors worldwide—a number expected to rise [1]. Despite these improvements, cancer survivors experience considerable side effects that negatively impact health-related quality of life (HRQoL), including fatigue, reduced physical function, and diminished cardiorespiratory fitness (CRF) [2–4]. Cancer survivors report lower HRQoL than the general population [2] and face a higher co-morbidity burden, with physical inactivity contributing [5]. Exercise is increasingly recognised as a cornerstone intervention for improving HRQoL, cancer-related fatigue, physical function, strength, and CRF among cancer survivors [6–8]. Furthermore, engaging in aerobic and resistance exercise has been shown to reduce the risk of chronic disease and protect against cancer-specific mortality [9, 10]. Current guidelines recommend that cancer survivors participate in a combination of aerobic and resistance exercise [11], yet many do not meet these recommendations [11, 12].

Evidence demonstrates that supervised exercise is superior in achieving improved health outcomes [7]; however, it is often infeasible due to barriers such as limited access to tailored facilities, travel, and time constraints [13, 14]. To overcome these barriers, supervised home-based exercise via videoconferencing is a promising solution [15]. This approach leverages known preferences such as tailored programmes, professional support, and exercising with other cancer survivors [13, 14]. Early studies on live-remote formats, including those that adapted to a live-remote online format during the COVID-19 pandemic, have demonstrated that they are safe, acceptable, and have high adherence [16–20]. Findings from these studies indicate improvements in HRQoL, fatigue, physical function, and CRF [17–19, 21], yet the impact of virtual exercise interventions on the cancer population remains limited [15].

We hypothesise that a live-remote exercise intervention will be superior to usual care in improving HRQoL, fatigue, and other physiological outcomes relevant to HRQoL in individuals treated for breast, prostate, and colorectal cancer. This study is one of the first large, purpose-designed randomised controlled trials (RCT) utilising this mode of exercise delivery in cancer survivors. The aim of this study was to compare the effects of EX-MED Cancer Sweden, a 12-week virtually supervised exercise intervention combining aerobic and resistance training, to a control group receiving usual care on HRQoL at 3 months, in people previously treated for breast, prostate, or colorectal cancer. Secondary aims were to compare the effects of the intervention’ on other patient-reported and physiological outcomes at 3 and 6 months.

## Methods

### Study design

The EX-MED Cancer Sweden trial (NCT05064670, www.clinicaltrials.gov) was a prospective superiority two-armed single-centre RCT [22]. Participants, who had completed curative treatment for cancer, were randomly allocated to participate in a 12-week live-remote exercise intervention (exercise group) or to receive usual care (control group). The primary outcome HRQOL and the secondary outcomes were measured at baseline, at 3 months (post-intervention) and at 6 months (follow-up). The intervention group commenced exercise training within 2 weeks of performing baseline measures. Reporting of the trial has been conducted in accordance with the CONSORT reporting guidelines [23].

### Setting and participants

Recruitment took place at Karolinska Comprehensive Cancer Centre (Stockholm, Sweden) between January 2022 and December 2023. Eligibility criteria were as follows: age ≥ 18 years, stage I–IIIa breast, prostate, or colorectal cancer, and within 5 years of completed curative treatment (those currently receiving endocrine therapy were eligible for participation). Individuals were excluded if they were performing regular exercise during the previous month (defined as at least ≥ 150 min moderate-intensity aerobic exercise and ≥ 2 structured resistance exercise sessions per week), had any medical conditions that may have prevented safe participation in the testing or exercise demands of the trial, or did not speak or understand the Swedish language.

### Randomisation and blinding

Participants were randomly allocated to either the exercise intervention group or the control group, following baseline measures, in a 1:1 ratio using a block size of four and stratified by cancer type and treatment with chemotherapy (yes/no), using a computer-generated randomisation programme. Study investigators and participants were blinded to group allocation at baseline, but not at the subsequent testing timepoints.

### Exercise intervention

Participants engaged in two 60-min live-remote exercise sessions per week for 12 weeks (24 sessions in total) in groups of up to eight. The intervention has been described in detail elsewhere [22]. In brief, the sessions combined both aerobic and resistance exercises (Supplementary tab 1). Each session was divided into four blocks, with each block containing three exercises—two strength and one aerobic. The exercises were performed in timed intervals, with each exercise followed by a short rest period before moving on to the next exercise. Sessions included a warm-up and cool down. Participants were encouraged to achieve an overall intensity of 14–18 on Borg’s Rating of Perceived Exertion (RPE) scale (range 6–20). This level allowed for accommodation of individual variation in fitness and symptom burden, while ensuring the overall exercise dose reflected a moderate-to-vigorous intensity [24, 25]. Intensity and load were manipulated using body weight, resistance bands (light and medium), and by varying the number of repetitions. Prior to commencing the exercise intervention, all participants had an in-person familiarisation with a member of the research team. During this session, participants received resistance bands, exercise programme details, and Microsoft® Teams setup guidance. Any barriers to participation were discussed and solutions found together.

The exercise sessions were delivered by personal trainers (*n* = 2) from a community-based gym, who had completed specialised exercise oncology education modules. The trainers supported the participants in achieving the intended exercise intensity and modified exercises according to individual response. A research team member joined the initial session for technical support. To maximise group cohesion, participants attended the same group and were given the option of morning, lunch, or evening sessions at set times. Participants were encouraged to engage in at least 150 min of moderate physical activity per week, inclusive of the intervention sessions. At the conclusion of the 12-week intervention, participants received a simple personalised physical activity prescription (physical activity on prescription, PAP) and had the opportunity to discuss their goals, preferences, and barriers to future exercise participation. The PAP allows registered health care professionals to prescribe physical activity outside of clinical settings with the aim of increasing physical activity levels and incorporating it into daily routines.

### Control group

Participants allocated to the control group received usual care as per national clinical care guidelines for cancer rehabilitation [26], which includes information and advice about physical activity from a health care professional but does not involve a structured exercise programme. After the 6-month follow-up, control group participants were also given a PAP and two home-exercise programmes (the intervention programme and a simpler one using sets and repetitions instead of timed intervals) for optional self-directed exercise.

### Outcome measures

All outcome measurements took place at baseline, 3 months (end of the intervention) and 6 months (3 months after the end of the intervention). Patient-reported outcomes were collected using online questionnaires prior to attending the study centre to complete physical assessments in person. Combined scores for questionnaires were calculated according to their corresponding scoring manual/recommendations.

### Primary outcome

Overall HRQoL at 3 months was assessed using the summary score of the Swedish version of the 30-item EORTC-QLQ-C30 questionnaire (version 3.0) [27], which consists of five functional scales (physical, role, emotional, cognitive, and social), three symptom scales (fatigue, nausea, and vomiting, and pain), six single items (dyspnoea, insomnia, appetite loss, constipation, diarrhoea, and financial difficulties), and a global HRQoL score (score range: 0–100). The summary score was calculated using 13 subscales, excluding the global QoL and financial difficulties items [28]. A higher score represents better HRQoL.

### Secondary outcomes

Secondary outcomes included the primary outcome assessed at 6 months, as well as a range of other patient-reported and physiological outcomes assessed at 3 and 6 months. Global HRQoL and all other functional and symptom scales and single items of the EORTC-QLQ-C30 were assessed, with higher scores representing better HRQoL or higher functioning for the global HRQoL score and functional scales, while higher symptom scores denote a greater level of symptoms. Cancer-related fatigue was also assessed using the Swedish version of the 22-item Piper Fatigue Scale (PFS) [29]. The PFS measures different dimensions of fatigue (behavioural, physical, cognitive, and emotional and total fatigue; score range: 1–10). Patient-reported physical activity was measured using a modified version of the Godin–Shephard Leisure-Time Physical Activity Questionnaire [30]. The Godin questionnaire consists of four items that ask about the average frequency and duration of mild-, moderate-, and vigorous-intensity aerobic exercise, as well as resistance exercise, performed in bouts of at least 10-min sessions during leisure time over a typical week in the past month. The time of the study intervention was reported in the Godin questionnaire by participants in the exercise group. Self-efficacy for exercise was assessed using the Swedish version of the Self-efficacy for Exercise (SEE-SV) questionnaire [31]. The SEE-SV measures an individual’s confidence in performing exercise despite various barriers and consists of nine items, score range 0–90, with a higher score indicating higher self-efficacy.

Physiological outcomes included physical function, CRF, muscle strength, and body composition. Physical function was assessed by the five times sit-to-stand test [32]. CRF, measured as estimated maximum oxygen uptake (VO_2 max_), was assessed by the Ekblom-Bak submaximal cycle test [33]. Muscle strength was assessed by the estimated 1 repetition maximum (1-RM) test for both the upper body (chest press) and lower body (leg press) [34]. Additionally, handgrip strength was assessed by a hydraulic hand dynamometer (SAEHAN Corporation, South Korea) [35]. Body composition was assessed using bio-impedance analysis (BIA) to assess body fat (BF%), fat mass (kg), and lean mass (kg), using the InBody770 (InBody Corporation, South Korea).

### Additional measures

Demographic and clinical information, including cancer treatments, were collected at baseline through questionnaires and from medical records. Adherence to the exercise intervention was measured as the percentage of the total number of planned exercise sessions attended. The personal trainer recorded attendance at each session. Intensity of exercise was measured by Borg’s RPE scale and recorded by the participants in a training logbook at the end of each session. Safety was assessed by the reporting of serious adverse events (SAEs) that occurred during the exercise and testing sessions by the personal trainer or the participants themselves. SAEs were defined as Grade 3 to 5 adverse events according to the Common Terminology Criteria for Adverse Events (CTCAE) [36].

### Statistical methods

An improvement in overall HRQOL in the exercise group from baseline to post-intervention at 3 months relative to the control group was of relevance. Based on previous exercise oncology trials using the EORTC-QLQ-C30, where small to moderate improvements were observed post-intervention [37], we calculated that a sample size of 80 participants per group was required to detect a medium effect size of 0.5 with 80% power and an alpha level of 0.05 (two-tailed). With an expected attrition rate of 20% [38], the target sample size was 200 participants (*n* = 100 per group). Data were analysed using IBM SPSS Statistics version 29 (IBM Corp. Released 2023. IBM SPSS Statistics for Windows, Version 29.0.2.0 Armonk, NY: IBM Corp). Numerical variables were summarised as mean and standard deviation (SD) or median and interquartile range (IQR) where more appropriate. Categorical variables were described as frequencies (proportions). Variable distributions were visually inspected by histograms. Linear mixed-effects models allowing for a random intercept were used to study change over time. The robust sandwich estimator was used to estimate standard errors robust to misspecifications of the covariance structure. Models were adjusted for the baseline value of the outcome, stratification factors (that is, cancer type and treatment with chemotherapy (yes/no), age, and sex. For all analyses, we used the intention-to-treat principle. Available data for participants with missing data were included under the missing at random assumption of the linear mixed-effects model. All data were checked for accuracy and screened for extreme values. Reported days of moderate and vigorous exercise exceeding 7 days were considered out of range values in the Godin Leisure Time Exercise Questionnaire and were excluded from the analysis (4 values). Additionally, moderate-to-vigorous *Z*-scores > 3.29 were considered univariate outliers and removed (8 values) [39]. Furthermore, we excluded the estimated VO_2 max_ values outside the valid range of the Ekblom-Bak cycle test (7 values) and those from individuals taking beta-blockers (13 values) [40]. No other values were excluded from the data. Adjustment for multiple groupwise comparisons was performed by using Bonferroni post-hoc corrections. Clinically important changes were estimated as standardised effect sizes (ES) where the adjusted between-group differences (BGD) post-intervention (3 months) and at follow-up (6 months) were divided by the pooled baseline standard deviation [41]. According to Cohen’s guidelines, effect sizes of 0–0.19, 0.2–0.49, 0.5–0.79, and > 0.8 were considered no to trivial, small, medium, and large effects, respectively [42].

As a sensitivity analysis, we repeated the linear mixed effects regression analyses using rank-transformed data for variables showing a heavily skewed distribution to test the robustness of our results. Additionally, we conducted a per-protocol analysis, excluding participants who did not adhere to the exercise intervention (defined as those attending < 75% of scheduled sessions) [43].

## Results

### Participant characteristics

**Fig. 1:**
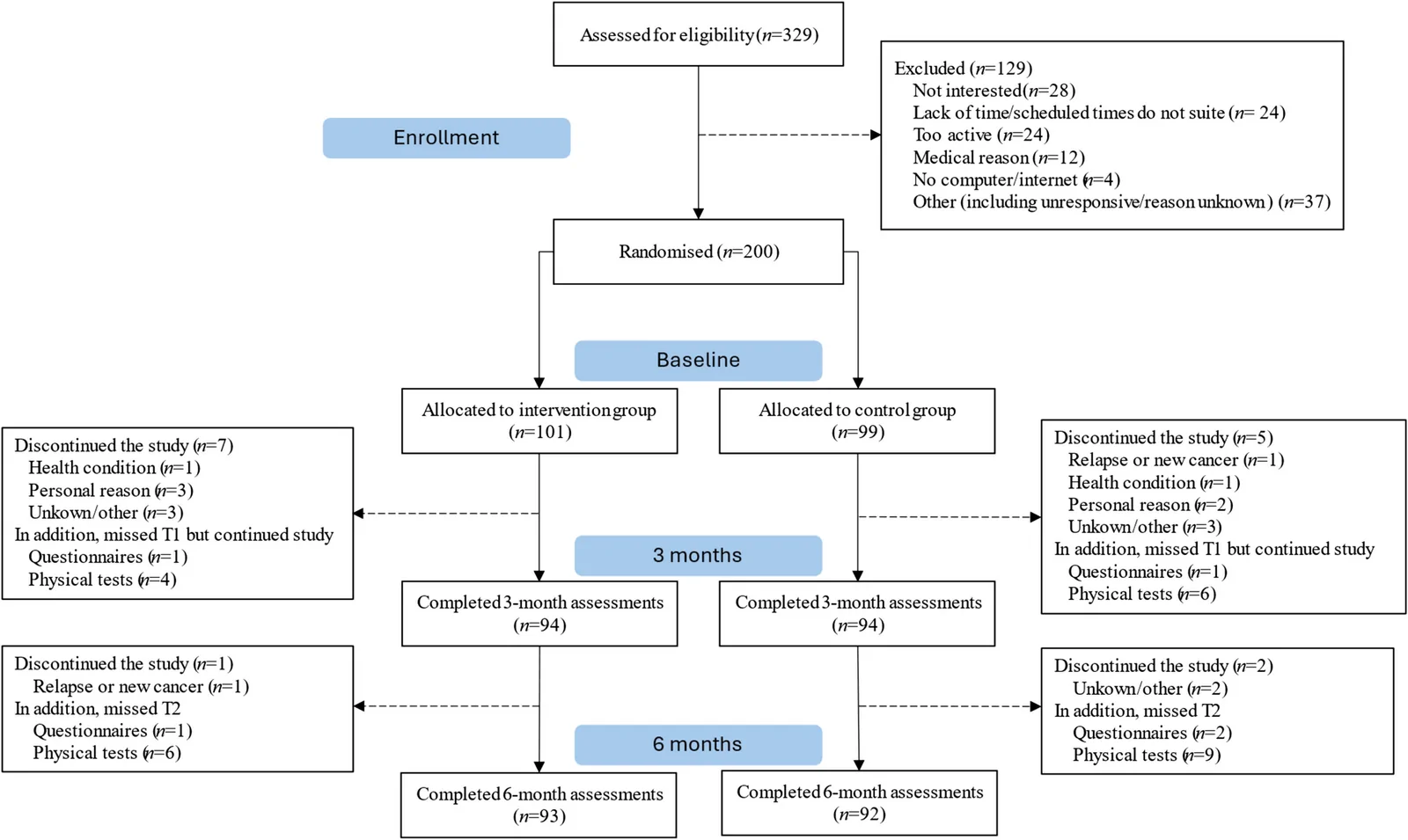
CONSORT diagram—flow of participants through the study

A total of 329 individuals were invited to participate in the study, of which 200 provided consent and were enrolled (recruitment rate, 60.8%), with 101 randomised to the exercise group and 99 to the control group (Fig. 1). The exercise and control groups had similar sociodemographic and clinical characteristics at baseline (Table 1). The mean age of the participants was 58.5 years (SD 10.4); 124 (62%) were female; 101 (50.5%) were treated for breast cancer; 53 (26.5%) for prostate cancer, and 46 (23%) for colorectal cancer. More than half of the participants (64%) were diagnosed less than 2 years prior to study enrolment, and about half (52%) had received chemotherapy as part of their treatment regimen. Twelve participants (6%) had dropped out at 3 months, and three (1.5%) had dropped out at 6 months. Participants’ characteristics were similar between those who withdrew and those who completed the study. At baseline, the median (interquartile range (IQR)) of patient-reported moderate-to-vigorous aerobic exercise minutes per week was 30 (0–120).

**Table 1:** Sociodemographic and clinical characteristics

	Exercise group *n* = 101	Control group *n* = 99
**Sociodemographic characteristics**		
Age (years, mean (SD))	58.5 (10.6)	58.5 (10.2)
Sex		
Female	61 (60.4)	63 (63.6)
Male	40 (39.6)	36 (36.4)
Marital status		
Married or partnered	78 (77.2)	76 (78.4)
Widowed	2 (2.0)	2 (2.1)
Single	21 (20.8)	19 (19.6)
Education		
Primary	8 (7.9)	6 (6.3)
Secondary	21 (20.8)	30 (31.3)
Tertiary	70 (69.3)	59 (61.5)
Other	2 (2.0)	1 (1.0)
Body mass index (kg/m^2^, mean (SD))	26.0 (3.9)	26.5 (4.3)
Current smokers	2 (2.0)	2 (2.0)
Employed	65 (64.4)	61 (62.2)
On sick leave	12 (11.9)	15 (15.3)
Physical activity (median (IQR) min/week)		
Aerobic exercise mod-vig (min/week)	30 (0–125)	20 (0–90)
Resistance exercise (min/week)	0 (0–20)	0 (0–30)
**Clinical characteristics**		
Type of cancer		
Breast	51 (50.5)	50 (50.5)
Prostate	27 (26.7)	26 (26.3)
Colorectal	23 (22.8)	23 (23.2)
Time since diagnosis		
≤ 2 years	68 (67.3)	60 (60.6)
> 2 years	33 (32.7)	39 (39.4)
Previous cancer treatment		
Chemotherapy	54 (53.5)	50 (50.5)
Surgery	92 (91.1)	97 (98.0)
Radiotherapy	58 (57.4)	48 (48.5)
Targeted therapy^a^	7 (6.9)	8 (8.1)
Current endocrine treatment	40 (39.6)	41 (41.4)

Data given as *n* (%) unless otherwise indicated

*SD* standard deviation, *IQR* interquartile range

^a^Includes both biologic agents, such as cyclin-dependent kinase 4 (CDK4) and CDK6 inhibitors, programmed cell death protein 1 (PD-1) inhibitors, as well as human epidermal growth factor receptor 2 (HER2)-targeted therapy

### HRQoL and fatigue

**Fig. 2:**
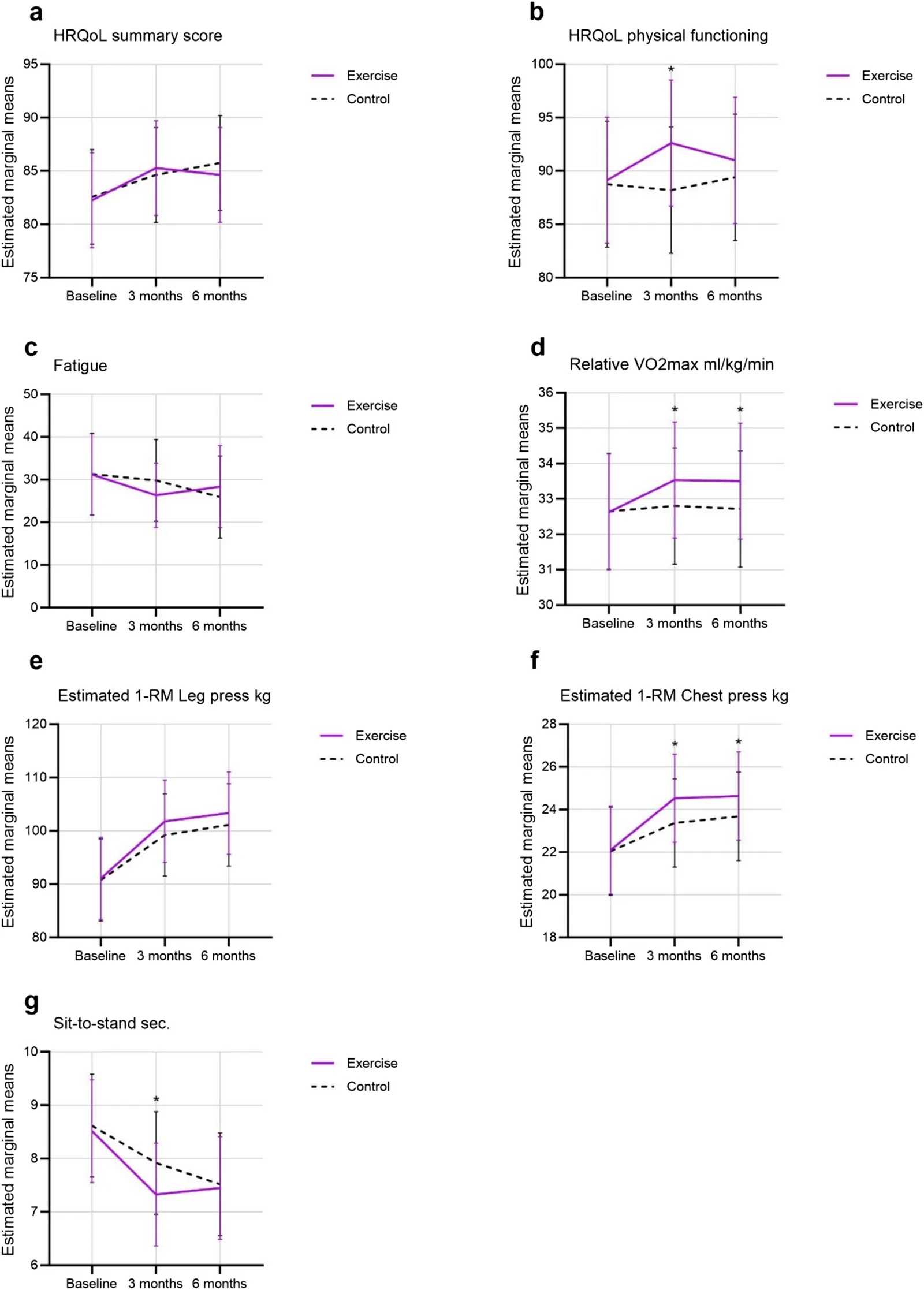
Changes in outcomes over the three timepoints in both the exercise and control groups. **a** HRQoL summary score, **b** HRQoL physical functioning, **c** fatigue, **d** relative VO_2 max_, **e** estimated 1-RM leg press, **f** estimated 1-RM chest press, and **g** 5 times sit-to-stand test. **a**–**c** Assessed by the EORTC-QLQ-C30 questionnaire. Data is presented as mean and standard error of the mean and based on the adjusted model. *Statistically significant BGD

Changes in overall HRQoL at 3 months, the study’s primary outcome, and at 6 months are shown in Table 2 and Fig. 2. There were no significant differences between the groups for overall HRQoL at 3 months, although the exercise group reported statistically significant better physical functioning on the QLQ-C30 scale than the control group at 3 months (BGD 4.41; 95% CI 2.05–6.77; *p* ≤ 0.001; effect size (ES), 0.31); however, the BGD was no longer significant at 6 months. No differences were found between the intervention and control groups at 3 or 6 months for the remaining functioning scales: emotional, role, cognitive, or social, nor on overall HRQoL at 6 months. Despite no BGDs at 3 months, overall HRQoL significantly improved for both groups, with a relative improvement from baseline of 3.66% in the exercise group and 2.50% in the control group (Supplementary tab 2).

**Table 2:** Effects of the EX-MED Sweden exercise intervention on HRQOL, fatigue, physical activity levels, and physiological outcomes

	**Arm**	**Unadjusted means**				**Adjusted between group differences**					
		**Baseline**	**3 months**	**6 months**		**BGDs at 3 months**			**BGDs at 6 months**		
		**Mean (SD)**	**Mean (SD)**	**Mean (SD)**		**Mean (95% CI)**	***p* value**	**ES**	**Mean (95% CI)**	***p* value**	**ES**
**EORTC QLQ-C30**											
Summary score	EX	81.93 (13.39)	85.09 (10.26)	84.08 (11.89)	EXvsCG	0.64 (− 1.15 to 2.42)	0.486	0.04	− 1.12 (− 2.92 to 0.68)	0.224	0.08
	CG	83.06 (13.66)	85.11 (12.79)	86.54 (11.59							
Global health status	EX	70.46 (19.38)	74.46 (17.63)	71.41 (20.46)	EXvsCG	1.93 (− 1.97 to 5.83)	0.332	0.10	− 2.26 (− 6.22 to 1.70)	0.263	0.12
	CG	67.87 (18.40)	70.79 (21.84)	72.75 (21.46)							
Physical functioning	EX	89.49 (12.34)	92.69 (8.96)	91.07 (13.16)	EXvsCG	4.41 (2.05–6.77)*	** < 0.001**	0.31	1.59 (− 0.81 to 3.99)	0.194	0.11
	CG	88.18 (16.23)	87.49 (15.77)	89.27 (14.65)							
Emotional functioning	EX	75.61 (24.62)	77.96 (20.40)	81.58 (20.84)	EXvsCG	− 2.04 (− 5.83 to 1.74)	0.289	0.09	− 2.92 (− 6.77 to 0.93)	0.136	0.13
	CG	78.26 (20.43)	76.52 (22.45)	81.55 (19.47)							
Role functioning	EX	81.36 (24.07)	87.46 (20.21)	84.96 (23.96)	EXvsCG	1.67 (− 2.84 to 6.18)	0.468	0.07	− 3.48 (− 8.06 to 1.11)	0.137	0.14
	CG	79.38 (25.43)	83.86 (22.86)	86.71 (20.28)							
Cognitive functioning	EX	75.42 (24.67)	78.14 (20.85)	78.50 (22.60)	EXvsCG	− 1.44 (− 5.06 to 2.19)	0.437	0.06	− 0.66 (− 4.34 to 3.03)	0.727	0.03
	CG	78.70 (25.99)	81.93 (21.83)	82.02 (22.36)							
Social functioning	EX	72.94 (26.55)	81.72 (21.43)	81.72 (21.99)	EXvsCG	− 1.87 (− 6.52 to 2.77)	0.429	0.07	− 0.71 (− 5.43 to 4.01)	0.767	0.03
	CG	74.15 (28.23)	84.21 (23.24)	83.15 (23.50)							
Fatigue	EX	30.69 (24.05)	26.46 (18.92)	28.67 (23.19)	EXvsCG	− 3.46 (− 7.29 to 0.37)	0.077	0.14	2.44 (− 1.45 to 6.33)	0.218	0.10
	CG	31.69 (24.23)	30.23 (26.06)	25.34 (22.78)							
Dyspnoea	EX	28.71 (23.11)	25.80 (23.11)	24.64 (26.09)	EXvsCG	− 1.57 (− 6.95 to 3.82)	0.568	0.06	0.08 (− 5.54 to 5.57)	0.976	0.00
	CG	28.18 (25.16)	27.27 (25.72)	24.24 (31.76)							
Insomnia	EX	33.99 (32.31)	27.24 (25.03)	29.71 (27.72)	EXvsCG	− 2.17 (− 7.42 to 3.09)	0.418	0.07	1.03 (− 4.33 to 6.38)	0.707	0.03
	CG	27.55 (31.76)	24.91 (28.34)	23.21 (24.32)							
**Fatigue** (Piper Fatigue Scale)											
Behaviour/daily life fatigue	EX	3.56 (2.66)	3.36 (2.39)	3.31 (2.33)	EXvsCG	− 0.16 (− 0.54 to 0.23)	0.425	0.06	− 0.18 (− 0.58 to 0.21)	0.364	0.07
	CG	3.36 (2.70)	3.41 (2.70)	3.27 (2.70)							
Emotional/affective fatigue	EX	4.43 (2.54)	4.28 (2.34)	4.40 (2.37)	EXvsCG	0.02 (− 0.39 to 0.42)	0.939	0.01	0.05 (− 0.36 to 0.46)	0.817	0.02
	CG	3.97 (2.69)	3.98 (2.75)	4.04 (2.79)							
Sensory/physical fatigue	EX	4.43 (2.43)	4.08 (2.22)	4.18 (2.33)	EXvsCG	− 0.07 (− 0.46 to 0.34)	0.745	0.03	0.11 (− 0.31 to 0.52)	0.617	0.04
	CG	4.27 (2.61)	4.05 (2.67)	3.85 (2.59)							
Cognitive fatigue	EX	3.75 (2.17)	3.51 (2.03)	3.57 (2.06)	EXvsCG	− 0.05 (− 0.39 to 0.29)	0.778	0.02	− 0.03 (− 0.37 to 0.31)	0.874	0.01
	CG	3.37 (2.30)	3.34 (2.32)	3.30 (2.25)							
Total fatigue	EX	4.02 (2.28)	3.80 (2.08)	3.90 (2.12)	EXvsCG	− 0.06 (− 0.40 to 0.27)	0.707	0.03	− 0.01 (− 0.35 to 0.33)	0.956	0.00
	CG	3.70 (2.43)	3.65 (2.49)	3.60 (2.45)							
**Self-efficacy of exercise** (Self-Efficacy of Exercise questionnaire)											
SEE score	EX	62.39 (23.23)	52.22 (24.40)	55.04 (24.79)	EXvsCG	− 0.67 (− 6.35 to 5.02)	0.817	0.03	4.33 (− 1.44 to 10.10)	0.141	0.18
	CG	52.89 (24.80)	46.78 (22.56)	44.83 (23.87)							
**Patient-reported physical activity** (Godin–Shephard Leisure-Time Physical Activity Questionnaire)											
Moderate-vigorous intensity aerobic exercise (min/week)	EX	88 (124)	152 (109)	118 (126)	EXvsCG	61 (31–90)*	** < 0.001**	0.57	2 (− 27 to 31)	0.893	0.02
	CG	61 (85)	74 (84)	99 (107)							
Resistance exercise (min/week)	EX	19 (41)	72 (57)	34 (37)	EXvsCG	38 (24–51)*	** < 0.001**	1.03	− 11 (− 25 to 2)	0.103	0.30
	CG	18 (32)	31 (44)	42 (59)							
**Cardiorespiratory fitness**											
Estimated VO2 max (ml/kg/min)	EX	32.56 (5.49)	32.95 (5.94)	33.13 (5.78)	EXvsCG	0.73 (0.02–1.44)*	**0.045**	0.12	0.78 (0.05–1.52)*	**0.036**	0.13
	CG	32.50 (6.23)	33.27 (5.98)	33.06 (6.61)							
**Physical strength and function**											
Leg press (kg)	EX	92.8 (25.0)	103.4 (25.7)	104.1 (25.7)	EXvsCG	2.6 (− 0.7 to 5.8)	0.123	0.10	2.2 (− 1.7 to 5.6)	0.199	0.09
	CG	87.7 (25.1)	97.3 (28.3)	99.4 (25.9)							
Chest press (kg)	EX	22.3 (9.9)	24.7 (9.9)	24.4 (10.3)	EXvsCG	1.2 (0.3–2.0)*	**0.010**	0.17	1.0 (0.1–1.8)*	**0.037**	0.11
	CG	21.0 (9.1)	22.9 (9.3)	23.6 (9.4)							
Handgrip right (kg)	EX	33.4 (11.9)	35.6 (11.9)	36.5 (12.9)	EXvsCG	0.7 (− 0.3 to 1.7)	0.162	0.06	0.5 (− 0.5 to 1.6)	0.313	0.04
	CG	32.5 (11.3)	34.6 (12.1)	35.0 (11.6)							
Handgrip left (kg)	EX	31.3 (11.7)	33.6 (11.8)	34.8 (12.3)	EXvsCG	0.9 (− 0.1 to 1.9)	0.081	0.08	0.9 (− 0.1 to 1.9)	0.093	0.08
	CG	30.7 (10.7)	32.7 (11.3)	34.1 (11.4)							
Sit to stand (sec. to complete 5 reps.)	EX	8.38 (2.27)	7.2 (2.0)	7.3 (2.3)	EXvsCG	− 0.59 (− 0.99 to − 0.2)*	**0.003**	0.22	− 0.07 (− 0.04 to 0.34)	0.743	0.03
	CG	8.83 (3.05)	8.0 (3.2)	7.7 (2.5)							
**Body composition**											
Body fat (%)	EX	30.1 (8.4)	30.4 (9.0)	30.2 (8.8)	EXvsCG	− 0.1 (− 0.6 to 0.4)	0.664	0.01	− 0.6 (− 1.1 to − 0.1)*	**0.016**	0.07
	CG	31.4 (8.4)	30.8 (8.2)	30.8 (7.9)							
Fat mass (kg)	EX	23.7 (9.3)	24.0 (10.0)	23.6 (9.5)	EXvsCG	0.0 (− 0.5 to 0.5)	0.945	0.00	− 0.7 (− 1.2 to − 0.1)*	**0.014**	0.07
	CG	24.9 (9.6)	24.3 (9.3)	24.3 (9.3)							
Lean mass (kg)	EX	29.7 (6.8)	29.5 (6.7)	29.2 (6.8)	EXvsCG	− 0.1 (− 0.3 to 0.1)	0.273	0.02	0 (− 0.2 to 0.2)	0.789	0.00
	CG	29.0 (6.1)	29.3 (6.1)	29.2 (6.0)							

Models were adjusted for the baseline value of the outcome, stratification factors (that is, cancer type and history of chemotherapy yes/no), age, and sex and included all available data

^*^Indicates significant differences (*p* < 0.05). Significant BGDs are in bold

*EX*, exercise group; *CG*, control group; *ES*, effect size according to Cohen

Only the symptoms of fatigue, dyspnoea, and insomnia of the QLQ-C30 were included in the analysis, as the majority of symptoms (nausea/vomiting, pain, appetite loss, constipation, diarrhoea, and financial problems) were infrequently reported in the study sample, with a median value of 0. The exercise group reported significantly decreased fatigue from baseline to post-intervention (− 4.85; 95% CI − 9.44 to − 0.25; *p* = 0.035) (Supplementary tab 2); however, when compared with the control group, there was no significant BGD found (BGD − 3.46; 95% CI 1.4–7.3; *p* = 0.077; ES 0.14). The control group also reported a decrease in fatigue, but this change was not statistically significant. No significant BGDs were found for either dyspnoea or insomnia. No BGDs were found for fatigue assessed by the Piper Fatigue Scale at either timepoint.

### Cardiorespiratory fitness, muscle strength, physical function, and body composition

Changes in CRF, muscle strength, physical function, and body composition are shown in Table 2 and Fig. 2. Positive exercise effects on CRF were observed at 3 months with significantly improved relative VO_2 max_ in the intervention group compared to the control group (BGD 0.73 ml/kg/min; 95% CI 0.02–1.44; *p* = 0.045; ES 0.12) which remained at 6 months (BGD 0.78 ml/kg/min; 95% CI 0.05–1.52; *p* = 0.036; ES 0.13).

Compared to the control group, the exercise group showed a significant improvement in upper body muscle strength with a significant increase in the estimated 1-RM chest press at 3 months (BGD 1.2 kg; 95% CI 0.3–2.0; *p* = 0.010; ES 0.17), which was maintained at 6 months (BGD 1.0 kg; 95% CI 0.1–1.8; *p* = 0.037; ES 0.11). At 3 months, the exercise group had significantly better physical function than the control group assessed by the sit-to-stand test (BGD − 0.59 secs; 95% CI − 0.99 to − 0.2; *p* = 0.003; ES 0.22); however, the BGD was no longer significant at 6 months. The BGDs for the estimated 1-RM leg press and handgrip strength favoured the intervention group although they did not meet the prespecified significance threshold.

With regard to body composition, trivial but significantly positive effects of the exercise intervention were seen only at 6 months on body fat percent and fat mass (BGD − 0.6%; 95% CI − 1.1 to − 0.1; *p* = 0.016; ES 0.07; and BGD − 0.7 kg; 95% CI − 1.2 to − 0.1; *p* = 0.014; ES 0.07). No BGDs were observed for lean mass at either timepoint.

### Patient-reported physical activity and self-efficacy for exercise

Compared to the control group, the exercise group significantly increased patient-reported moderate-vigorous intensity aerobic exercise levels at 3 months (BGD 61 min per week; 95% CI 31–90; *p* ≤ 0.001; ES 0.57), as well as resistance exercise levels (BGD 38 min per week; 95% CI 24–51; *p* ≤ 0.001; ES 1.03). However, at 6 months, aerobic and resistance exercise levels significantly improved for the control group, as well as resulting in a lack of significant differences between the groups (Supplementary tab 2). No beneficial effects of the exercise intervention were found on self-efficacy for exercise at either timepoint.

Within-group differences for all outcomes are shown in Supplementary Table 2.

### Safety and adherence

No serious adverse events (SAEs) were reported as a result of the exercise sessions or physical testing. The median (IQR) attendance rate for supervised exercise sessions was 75% (67–88%). The main reasons given for non-attendance were illness, travel or holidays, and work commitments; in many cases, reasons were unspecified. The median overall exercise intensity level for all exercise sessions according to Borg’s RPE scale was 14 [13–15], reflecting a moderate intensity, with 52% of the participants able to achieve an average intensity of 14 or above.

### Sensitivity analyses

The sensitivity analysis, using rank-transformed variables for skewed data, yielded similar results. A per-protocol analysis excluding the 40 participants (39.6%) who did not adhere to the exercise intervention (that is, attending < 75% of scheduled exercise sessions) supported the findings of the intention-to-treat analysis, with similar effect estimates and statistical significance for most of the outcomes (Supplementary tab 3). However, for relative VO_2 max_ at 3 months, the exercise group demonstrated a significantly greater BGD compared to the control group, with a larger effect size and stronger statistical significance in those who attended ≥ 75% of the scheduled exercise sessions. Additionally, the estimated 1-RM leg press, which was not statistically significant in the intention-to-treat analysis, was significantly different at 3 months.

## Discussion

The EX-MED Cancer Sweden RCT investigated the effects of a live-remote exercise intervention compared to usual care on various patient-reported and physiological outcomes in cancer survivors post-treatment. Our findings indicate that participating in a 12-week live-remote exercise intervention, conducted in group-based sessions, led to significant improvements in physical function, CRF, and muscle strength. However, no significant changes were observed on overall HRQoL. This is in contrast to previous reviews and meta-analyses that have reported small to moderate improvements in cancer survivor’s HRQoL following exercise interventions [37, 44, 45]. In our study, the lack of intervention effects on HRQoL may be explained by notable ceiling and floor effects [46], as many participants reported high levels of HRQoL and functioning and were largely free of symptoms at baseline. Compared to the general Swedish population, participants in our study reported similar HRQoL levels [47], suggesting limited room for further improvement. Furthermore, participants were enrolled up to 5 years post-treatment. During this survivorship period, HRQoL may improve through natural recovery and adaptation to life post-cancer, particularly in older cancer survivors [48, 49]. This may have diminished the potential effect of the exercise intervention.

Notably, we observed statistically significant improvements in the physical functioning domain of HRQoL, as well as in objectively measured physical function (sit-to-stand test) at 3 months. Although the effect sizes were small, they are consistent with those reported in other exercise oncology trials, which often exhibit small to moderate effect sizes [6, 8, 37]. These improvements are clinically relevant for cancer survivors, as physical function encompasses essential activities such as self-care, mobility, and physical exercise, which are key determinants of HRQoL [50]. Poor physical function is likely to lead to poor HRQoL. This is particularly important as more people are surviving cancer and living longer with previous studies demonstrating a strong association between physical function and HRQoL in older adults [51, 52]. Moreover, the prevalence of patient-reported functional limitations among cancer survivors has increased, with those under 65 experiencing the greatest rise in limitations [4].

In addition to improvements in physical function, we also observed significant yet small improvements in upper body chest strength at 3 and 6 months, although improvements in lower body leg and grip strength were not significant. Previous home-based exercise studies have lacked consistent improvements in muscle strength among cancer survivors [44, 53, 54] compared to in-person studies [8, 55]. Interestingly, smaller RCTs of home-based exercise but with face-to-face virtual instruction, similar to our study, reported significantly positive effects on strength in breast cancer survivors, both during treatment and post-treatment [56, 57]. These results suggest the importance of active supervision in the home-based setting to support correct technique and safety, as resistance exercises are often more complex, and participants may lack confidence in performing them. One possible explanation for the observed improvements in upper body chest strength but not in lower body leg strength in our study could be the use of resistance bands for upper body exercises but no equipment to additionally load the lower body muscles. This highlights the need for future interventions to carefully consider and ensure an appropriate load when exercising in a home-based setting to achieve improvements in both upper and lower body strength, e.g. using dumbbells or mini loop resistance bands around the legs.

Significant improvements in CRF were demonstrated at 3 and 6 months, however with a very small effect size. In contrast, previous exercise trials in cancer survivors have shown significant moderate to large improvements in CRF [37, 45]. However, a recent meta-analysis in breast and prostate cancer survivors found no significant effect of unsupervised, home-based exercise on CRF [44]. Similarly, Lahart et al. reported greater CRF gains with supervised exercise programmes compared to unsupervised ones in breast cancer survivors [37], suggesting that supervision may be an important factor in improving CRF. Additionally, studies of high intensity interval training (HIIT) exercise in cancer survivors have demonstrated significantly large improvements in CRF [58].

In our study, only 52% of participants achieved the target intensity (RPE 14–18) despite supervision, leading us to speculate that greater adherence to the prescribed intensity would have led to even more pronounced effects. Interestingly, two exercise studies that had cohorts who transitioned to live-remote delivery during the COVID-19 pandemic reported that approximately one fifth of participants found the exercise intensity easier in the virtual sessions compared to in-person sessions [18, 20]. Altogether, this suggests that achieving sufficient exercise intensity to improve CRF may be challenging in home-based settings, even with virtual supervision. Recommendations to use a bike or treadmill [58] may not be feasible in a home-based setting; however, equipment such as a stepper that is less costly and takes up less space could be considered. While the clinical relevance of our findings should be interpreted with caution, they highlight the potential challenges of achieving sufficient exercise intensity to induce changes in CRF in remotely delivered programmes. However, strategies to improve CRF remain highly relevant as cancer survivors typically have lower CRF levels [3], with poor CRF being linked to impaired HRQoL [59] as well as an increased risk of cancer-related and overall morbidity and mortality [60].

In concordance with other studies [61, 62], our study demonstrated significant improvements in physical function and certain physiological outcomes; however, there were no improvements in the emotional, role, cognitive, or social functioning domains of the EORTC-QLQ-C30. Although participants exercised in small groups together with other cancer survivors, the virtual setting limited the in-person interaction with both peers and the personal trainer. This reduced social engagement may explain the lack of impact on mental wellbeing. Moreover, the nature of exercise interventions focuses on physical outcomes; therefore, future interventions may need to incorporate additional components to improve psychological functioning [61, 63]. Evidence from previous research suggests that interventions such as cognitive behavioural therapy and mindfulness can enhance emotional wellbeing, cognitive, and social functioning [64, 65]. Furthermore, the high baseline psychological functioning of participants in our study suggests possible ceiling effects, which may have limited the potential to observe further improvements. Likewise, low baseline levels of cancer-related fatigue, insomnia, and dyspnoea point to floor effects, leaving limited room for improvement. In the case of fatigue, the lack of significant improvement observed may also be linked to the modest gains in CRF. Previous studies have shown that higher intensity exercise can improve fatigue in cancer survivors, with these benefits being mediated by improvements in CRF [66, 67]. This further reinforces the need to explore practical and scalable ways to facilitate higher intensity training in remote settings.

In the present study, the attendance rate of 75% aligns with previous research on home-based exercise interventions for cancer survivors, which reported attendance rates between 71 and 88% [53]. It also compares favourably with in-person interventions, with attendance rates between 65 and 77% [68–70], suggesting acceptance of the live-remote format and high participant motivation. Although there were no significant differences between the groups regarding social functioning, we believe that the social aspect of the intervention—specifically the presence of other survivors and the trainer during sessions—motivated participants to attend training sessions [71, 72]. Notably, our trial began recruitment approximately 20 months into the COVID-19 pandemic, a time when opportunities for social connection remained limited. This context may have further motivated participants to attend the live-remote sessions. Supporting this, our study achieved a high recruitment rate of 60.6%, which is considerably higher than the 38% reported in previous trials [73]. One of the main reasons for non-participation in these earlier trials has been travel constraints—an issue largely overcome by the live-remote format. Given that most cancer survivors report a preference for exercising at home [74] and that supervised exercise has been shown to yield superior health benefits [7], these findings underscore the potential benefit of the live-remote format.

No SAEs occurred during the exercise sessions or testing of the current study. However, it is essential to continue to develop strategies to ensure the safety of participants during virtual exercise sessions [75]. Strategies utilised in our study included screening for medical conditions, an in-person familiarisation session, the personal trainer knowing where participants were training remotely, and adapting exercises as needed.

In the current study, we found no improvements in self-efficacy for exercise. As observed in a previous study [76], self-efficacy was highest at baseline. This lack of improvement may explain why participants in the exercise group did not maintain their physical activity levels after the intervention was completed. Despite current guidelines recommending a 12-week intervention [11], we speculate that a longer intervention or a tapering period following the 12 weeks of supervision with additional support might have enhanced self-efficacy and supported the transition from supervised to unsupervised settings. However, Kampshoff et al. previously found that self-efficacy could not be established as a determinant of exercise maintenance [77].

This study is one of the first large RCTs investigating the effect of exercise delivered in a supervised virtual format on HRQoL and other relevant outcomes in cancer survivors. While the live-remote exercise intervention did not lead to improvements in overall HRQoL, it had a positive impact on physical function, CRF, and muscle strength, all of which are important aspects of HRQoL and healthy cancer survivorship. Our study strengthens the early evidence from previous trials regarding the efficacy and feasibility of supervised virtual exercise programmes. However, their effect on HRQoL remains unclear and warrants further investigation, particularly given that group-based live-remote delivery requires fewer resources, offers broader reach, and retains the crucial element of supervision. This mode of exercise support holds promise as an equitable strategy to making exercise programmes accessible to people affected by cancer, no matter where they live.

Our study is not without limitations. The population recruited was predominantly female, highly educated, and mostly treated for breast cancer. Additionally, we lack detailed information on the cancer survivors who declined participation, which may limit the generalisability of our results. Furthermore, the inherent challenge that individuals motivated to exercise are more likely to participate in exercise trials could have influenced our findings. As physical activity levels also increased in the control group, it is likely that our study population was already motivated to exercise, potentially affecting the intervention’s impact. While outcome assessors were blinded to group allocation at baseline, they could not be blinded at subsequent testing timepoints. Finally, while the Ekblom-Bak submaximal cycle test is a pragmatic, safe, and low-burden method for estimating VO_2 max_, it may lack sensitivity to detect training-induced changes—particularly in post-treatment cancer survivors [40]—which could have attenuated the observed effects of the intervention.

## Conclusion

Overall, this RCT found no significant effect of a supervised live-remote exercise intervention on cancer survivors’ HRQoL; however, the intervention did lead to beneficial effects on physical function, physical activity levels, CRF, and upper body strength—outcomes relevant to HRQoL and healthy survivorship. Given the potential to improve accessibility to tailored exercise programmes for cancer survivors while requiring fewer resources, implementation-effectiveness studies are warranted to further investigate the impact of live-remote exercise on HRQoL and to establish this format as an effective method of exercise delivery.

## Supplementary Information

Below is the link to the electronic supplementary material.

Supplementary file1 (PDF 226 KB)

## Data Availability

The datasets used during the current study are available from the corresponding author on reasonable request.

## Abbreviations

HRQoL: Health-related quality of life
CRF: Cardiorespiratory fitness
RCT: Randomised controlled trial
EORTC-QLQ-C30: European Organisation for Research and Treatment of Cancer Quality of Life Questionnaire
RPE: Rating of perceived exertion
PAP: Physical activity on prescription
SAE: Serious adverse events
1-RM: One repetition maximum
BIA: Bio-impedance analysis
ES: Effect size
BGD: Between-group difference
IQR: Interquartile range
