# Supplementary material for: Effects of live-remote exercise on quality of life and other health-related outcomes in cancer survivors: a randomised controlled trial
Source: J Cancer Surviv. 2025 Jun 5;20(Suppl 1):476–91. doi: 10.1007/s11764-025-01845-x (PMC13676532; doi:10.1007/s11764-025-01845-x)
Supplement: Supplementary file 1 [file 11764_2025_1845_MOESM1_ESM.pdf]

Supplementary table 1 The EX-MED Cancer Sweden exercise program

**Program structure[a]:** You will combine both aerobic and resistance exercises in the same session. Each session consists of four blocks with three exercises per block, 2 strength/1 aerobic. You will need a resistance band. Aim for an overall RPE of 14-18 according to the Borg's scale (6-20).			
**Structure[b]**	**Exercises[c]**	**Time**	**Comments**
**Block 1**	1. Squat with shoulder press	40 sec each, rest 20 sec<br>3 rounds, rest 1 min between each round<br>2 min rest before block 2	Place resistance band under feet for shoulder press
	2. Hip raise		
	3. High knees		
**Block 2**	1. Reverse lunge with one-arm row	40 sec each, rest 20 sec<br>3 rounds, rest 1 min between each round<br>2 min rest before block 3	Attach the resistance band to something sturdy like a door
	2. Core rotation		Attach the resistance band to something sturdy like a door
	3. Skaters		
**Block 3**	1. Single-leg deadlift	40 sec each, rest 20 sec<br>3 rounds, rest 1 min between each round<br>2 min rest before block 4	
	2. Forward lunge with chest press		Attach the resistance band to something sturdy like a door
	3. Cross-country skiing		
**Block 4**	1. Push-up	20 sec each, rest 10 sec<br>3 rounds, rest 1 min between each round	Can be done on knees, toes, against a bench or standing against a wall
	2. Back extension with lat pull		
	3. Squat with side stretch		
**Compliment this program with other physical activity at moderate intensity, such as brisk walking, cycling, swimming, so that you reach at least 150 minutes per week.**			

RPE Rating of Perceived Exertion
[a]The exercise program consisted of two supervised sessions of 60 minutes per week.
[b]All sessions started with a 5-minute warm-up and concluded with a 5-minute stretch.
[c]Variations of the exercises were allowed and the personal trainer made adaptions based on individual response. Intensity and load were manipulated using body weight, resistance bands (light and medium), and by varying the number of repetitions. The original program was complimented with photos of each exercise and the Borg RPE scale.

Supplementary table 2 Within-group differences in HRQOL, fatigue, physical activity levels and physiological outcomes

		Adjusted within group differences			
		WGDs Baseline to 3 months		WGDs Baseline to 6 months	
		Mean (95% CI)	p value	Mean (95% CI)	p value
**EORTC QLQ-C30**					
Summary score	EX	3.01 (0.88-5.14)*	0.002	2.37 (0.38-4.37)*	0.015
	CG	2.06 (0.04-2.09)*	0.044	3.179 (1.00-5.36)*	0.002
Global health status	EX	4.25 (-0.43-8.93)	0.089	1.16 (-2.67-4.99)	0.551
	CG	2.82 (-1.58-7.22)	0.300	3.93 (-0.85-8.71)	0.147
Physical functioning	EX	3.48 (0.64-6.31)*	0.010	1.86 (-0.80-4.51)	0.232
	CG	-0.55 (-3.10-1.99)	1.000	0.65 (-2.06-3.36)	1.000
Emotional functioning	EX	2.16 (-2.38-6.70)	0.762	1.54 (-2.72-5.79)	0.834
	CG	3.31 (-1.09-7.72)	0.197	3.57 (-1.08-8.21)	0.197
Role functioning	EX	5.65 (0.24-11.07)*	0.037	3.40 (-1.66-8.47)	0.263
	CG	4.27 (-0.64-9.57)	0.099	7.364 (1.81-12.91)*	0.005
Cognitive functioning	EX	2.67 (-1.38-6.77)	0.275	3.89 (-0.46-8.24)	0.096
	CG	3.93 (-0.70-7.48)	0.125	3.81 (-0.64-7.48)	0.120
Social functioning	EX	8.44 (2.96-13.93)*	0.001	8.50 (2.93-14.08)*	0.001
	CG	9.71 (4.11-15.31)*	0.000	8.61 (3.28-13.95)*	0.001
Fatigue	EX	-4.85 (-9.44 to -0.25)*	0.035	-2.84 (-7.14-1.46)	0.276
	CG	-1.51 (-5.29-2.26)	0.432	-5.41 (-10.10 to -0.71)*	0.018
Dyspnoea	EX	-3.03 (-9.08-3.02)	0.522	-4.19 (-10.67-2.30)	0.364
	CG	-1.27 (-6.59-4.06)	0.641	-4.07 (-10.70-2.55)	0.422
Insomnia	EX	-6.07 (-12.36-0.23)	0.063	-3.89 (-9.80-2.02)	0.279
	CG	-1.75 (-7.67-4.17)	1.000	-2.77 (-9.20-3.67)	0.908
**Fatigue** (Piper fatigue scale)					
Behaviour/daily life fatigue	EX	-0.20 (-0.63-0.23)	0.584	-0.36 (-0.79-0.12)	0.234
	CG	-0.01 (-0.39-0.37)	1.000	-0.12 (-0.59-0.36)	1.000
Emotional/affective fatigue	EX	-0.19 (-0.67-0.29)	1.000	-0.13 (-0.57-0.32)	1.000
	CG	-0.13 (-0.60-0.35)	1.000	-0.10 (-0.53-0.34)	1.000
Sensory/physical fatigue	EX	-0.35 (-0.84-0.14)	0.256	-0.29 (-0.75-0.17)	0.305
	CG	-0.24 (-0.70-0.22)	0.479	-0.35 (-0.85-0.14)	0.266
Cognitive fatigue	EX	-0.21 (-0.59-0.17)	0.446	-0.24 (-0.64-0.16)	0.466
	CG	-0.09 (-0.45-0.28)	1.000	-0.14 (-0.55-0.27)	1.000
Total fatigue	EX	-0.22 (-0.61-0.17)	0.503	-0.23 (-0.63-0.17)	0.503
	CG	-0.10 (-0.47-0.27)	1.000	-0.17 (-0.57-0.24)	0.982
**Self-efficacy of exercise** (Self-Efficacy of Exercise questionnaire)					
SEE score	EX	-10.03 (-16.78 to -3.28)*	0.001	-7.04 (-13.36 to -0.73)	0.301
	CG	-5.62 (-11.95-0.71)	0.093	-7.63 (-14.51 to -0.75)	0.489
**Patient-reported physical activity** (Godin–Shephard Leisure-Time Exercise Questionnaire)					
Moderate-vigorous intensity aerobic exercise (min/week)	EX	64 (30-98)*	<0.001	26 (-2-53)	0.071
	CG	15 (-15-45)	0.371	35 (0-70)*	0.050
Resistance exercise (min/week)	EX	51 (36-67)*	<0.001	10 (-3-22)	0.124

	CG	14 (1-29)*	0.049	22 (6-38)*	0.003
**Cardiorespiratory fitness**					
Estimated VO2max (ml/kg/min)	EX	0.90 (0.08-1.72)*	0.026	0.87 (0.09-1.67)*	0.026
	CG	0.15 (-0.71-1.01)	1.000	0.07 (-0.67-0.8)	1.000
**Physical strength and function**					
Leg press (kg)	EX	10.7 (7.2-14.3)*	<0.001	12.3 (8.4-16.1)*	<0.001
	CG	8.5 (4.9-12.0)*	<0.001	10.4 (6.4-14.3)*	<0.001
Chest press (kg)	EX	2.4 (1.5-3.4)*	<0.001	2.5 (1.5-3.6)*	<0.001
	CG	1.3 (0-2)*	0.004	1.6 (0.6.2.7)*	0.001
Handgrip right (kg)	EX	1.9 (0.8-3.1)*	<0.001	3.2 (2.0-4.4)*	<0.001
	CG	1.3 (0.3-2.3)*	0.012	2.7 (1.5-4.0)*	<0.001
Handgrip left (kg)	EX	2.1 (1.0-3.6)*	<0.001	3.6 (2.5-4.8)*	<0.001
	CG	1.2 (0.3-2.2)*	0.012	2.8 (1.6-4.0)*	<0.001
Sit to stand (sec. to complete 5 reps.)	EX	-1.19 (-1.65 to -0.72)*	<0.001	-1.06 (-1.51 to -0.62)*	<0.001
	CG	-0.70 (-1.1 to -0.26)*	0.001	-1.10 (-1.58 to -0.62)*	<0.001
**Body composition**					
Body fat (%)	EX	0.03 (-0.45-0.51)	1.000	-0.12 (-0.64-0.40)	1.000
	CG	0.14 (-0.33-0.61)	0.57	0.49 (-0.10-1.08)	0.145
Fat mass (kg)	EX	0.08 (-0.46-0.60)	1.000	-0.15 (-0.71-0.41)	1.000
	CG	0.06 (-0.43-0-56)	0.803	0.51 (-0.11-1.14)	0.141
Lean mass (kg)	EX	-0.17 (-0.38-0.03)	0.124	-0.06 (-0.23-0.11)	0.464
	CG	-0.08 (-0.28-0.13)	1.000	-0.09 (-0.23-0.13)	1.000

EX, exercise group; CG, control group. Models were adjusted for the baseline value of the outcome, stratification factors (that is, cancer type and history of chemotherapy yes/no), age and sex, and included all available data. *Indicates significant differences ($P < 0.05$).

Supplementary table 3 Per-protocol-analysis of the effects of the EX-MED Sweden exercise intervention on HRQOL, fatigue, physical activity levels and physiological outcomes

		Adjusted between group differences Per-protocol-analysis			
		**BGDs at 3 months**		**BGDs at 6 months**	
		**Mean (95% CI)**	**p value**	**Mean (95% CI)**	**p value**
**EORTC QLQ-C30**					
Summary score	EXvsCG	0.57 (-1.51-2.64)	0.592	-1.23 (-3.32-0.86)	0.247
Global health status	EXvsCG	3.29 (-1.31-7.88)	0.161	-1.79 (-6.44-2.87)	0.451
Physical functioning	EXvsCG	**4.59 (1.81-7.37)***	**0.001**	1.48 (-1.33-4.23)	0.302
Emotional functioning	EXvsCG	-3.00 (-7.29-1.30)	0.171	-2.69 (-7.04-1.69)	0.226
Role functioning	EXvsCG	2.64 (-2.30-7.56)	0.294	-4.36 (-9.36-0.65)	0.088
Cognitive functioning	EXvsCG	0.26 (-3.41-3.92)	0.890	-0.46 (-4.17-3.25)	0.809
Social functioning	EXvsCG	-1.56 (-6.73-3.61)	0.553	-1.03 (-6.26-4.21)	0.700
Fatigue	EXvsCG	-4.40 (-8.88-0.09)	0.055	1.42 (-3.13-5.96)	0.541
Dyspnoea	EXvsCG	-0.86 (-7.12-5.41)	0.788	-0.52 (-6.90-5.86)	0.873
Insomnia	EXvsCG	-0.41 (-6.42-5.61)	0.894	0.61 (-5.48-6.70)	0.844
**Fatigue** (Piper fatigue scale)					
Behaviour/daily life fatigue	EXvsCG	-0.04 (-0.50-0.41)	0.852	0.05 (-0.51-0.41)	0.830
Emotional/affective fatigue	EXvsCG	0.16 (-0.31-0.64)	0.527	0.12 (-0.36-0.60)	0.603
Sensory/physical fatigue	EXvsCG	-0.01 (-0,50-0.47)	0.959	0.09 (-0.39-0.58)	0.705
Cognitive fatigue	EXvsCG	0.01 (-0.38-0.40)	0.970	-0.07 (-0.46-0.32)	0.723
Total fatigue	EXvsCG	0.04 (-0.36-0.44)	0.851	0.03 (-0.36-0.44)	0.878
**Self-efficacy of exercise** (Self-Efficacy of Exercise questionnaire)					
SEE	EXvsCG	1.91 (-4.50-8.32)	0.558	5.58 (-0.94-12.01)	0.093
**Patient-reported physical activity** (Godin–Shephard Leisure-Time Physical Activity Questionnaire)					
Moderate-vigorous intensity aerobic exercise (min/week)	EXvsCG	**68 (36-100)***	**<0.001**	14 (-17-46)	0.373
Resistance exercise (min/week)	EXvsCG	**22 (7-37)***	**0.004**	-12 (27-3)	0.114
**Cardiorespiratory fitness**					
Estimated VO2max (ml/kg/min)	EXvsCG	**1.09 (0.26-1.91)***	**0.010**	**0.93 (0.08-1.77)***	**0.033**
Estimated VO2max (L/min)	EXvsCG	0.05 (-0.02-0.12)	0.150	0.04 (-0.03-0.11)	0.293
**Physical strength and function**					
Leg press (kg)	EXvsCG	**3.9 (0.1-7.6)***	**0.044**	2.3 (-1.5-6.2)	0.246
Chest press (kg)	EXvsCG	**1.4 (0.4-2.4)***	**0.006**	1.0 (0.0-2.0)	0.051
Handgrip right (kg)	EXvsCG	0.7 (-0.4-1.9)	0.203	0.6 (-0,6-1.8)	0.304
Handgrip left (kg)	EXvsCG	0.9 (-0.2-2.0)	0.094	1.1 (0.0-2.2)	0.052
Sit to stand (sec. to complete 5 reps.)	EXvsCG	**-0.6 (-1.1 to -0.2)***	**0.006**	-0.1 (-0.5-0.4)	0.802
**Body composition**					
Body fat (%)	EXvsCG	-0.3 (-0.9-0.2)	0.234	**-1.0 (-1.6 to -0.5)***	**<0.001**
Fat mass (kg)	EXvsCG	-0.2 (-0.8-0.3)	0.421	**-1.2 (-1.7 to -0.6)***	**<0.001**
Lean mass (kg)	EXvsCG	-0.1 (-0.3-0.1)	0.379	0.0 (-0.2-0.2)	0.846

EX, exercise group; CG, control group. Models were adjusted for the baseline value of the outcome, stratification factors (that is, cancer type and history of chemotherapy yes/no), age and sex, and included all available data. *Indicates significant differences ($P < 0.05$). Significant BGDs are in bold.
